# The prognostic and immunological role of NADPH oxidase in early and locally advanced lung adenocarcinoma

**DOI:** 10.1007/s12672-026-05404-3

**Published:** 2026-06-10

**Authors:** Jingjing Deng, Linfeng Cao, Gouxin Hou, Xiaolong Ma

**Affiliations:** 1 Department of Critical Care Medicine, the Key Clinical Specialties of Jiangsu Province, Yixing People’s Hospital, The Affiliated Yixing Hospital of Jiangsu University, Yixing Branch of Wuxi Medical Center of Nanjing Medical University, The Affiliated Yixing Clinical School of Medical School of Yangzhou University, No.1588 Xincheng Road, Yixing, 214200 Jiangsu China; 2 Department of Pulmonary and Critical Care Medicine, The First Hospital of Jiaxing, Affiliated Hospital of Jiaxing University, No. 1882 Zhonghuan South Road, Jiaxing, 314000 Zhejiang P.R. China; 3 Department of Oncology, The First Hospital of Jiaxing, Affiliated Hospital of Jiaxing University, Jiaxing, China

## Abstract

Lung adenocarcinoma (LUAD) is the pathological type with the highest proportion and heterogeneity in lung cancer. NADPH oxidase is an important enzyme in the biological respiratory chain that plays a decisive role in cell growth, regulation, and apoptosis, which translating seven gene families, namely *NOX1-5* and *DUOX1-2*. The current study aimed to investigate the expression, and prognostic and immune-related roles of this gene family in early and advanced LUAD. Gene expression was examined using the Oncomine™ database, prognostic value was analyzed by Kaplan-Meier analysis, expression of NOX2 and NOX4 in early and locally advanced LUAD was validated using RT-qPCR, and the correlation of infiltrating immune cells was validated by tumor immune estimation resources (TIMER). Over-expressed NOX4 was associated with lower prognosis; NOX2 was found to be under-expressed in LUAD. Both NOX2 and NOX4 expression was positively related to tumor purity and had positive correlation with infiltrating levels of immune cells in TIMER. The data reflected the potential association of NADPH oxidase in early and locally advanced lung adenocarcinoma. Our data suggested that the combination of NOX4 suppression, NOX2 depression, and immunotherapy would improve clinical outcome in these tumors.

**Supplementary Information:**

The online version contains supplementary material available at 10.1007/s12672-026-05404-3.

## Introduction

Among malignant tumors, lung cancer is the most common cancer [1, 2], non-small cell lung cancer (NSCLC), accounting for more than 80% of all cases of lung cancer [3]. Lung adenocarcinoma (LAUD) is the major and aggressive subtype of NSCLC, with the highest heterogeneity [4–6]. According to the JACC (American Joint Committee on Cancer) guidelines [7], early-stage NSCLC refers to stages I and II, and stage III, also called “locally advanced disease”, refers to a potentially resectable or unresectable tumor. Currently, with the development of physical examination methods, application of low-dose CT (LDCT) screening programs [8, 9] and artificial intelligence (AI) applications [10, 11] in populations at risk, early diagnosis of lung cancer has become possible. Although the diagnosis and treatment have dramatically improved, five-year survival rates in stage III of LUAD are still less than 50% [12] with minimal improvement from adjuvant chemotherapy [13]. Some studies [14] have revealed that immunotherapeutic agents are most effective at low tumour burden and with initial adjuvant chemotherapy. Forde et al. [15] showed that using anti-PD-1 monoclonal antibody blockers for neoadjuvant immunotherapy could improve the resectable lesions of NSCLC. Therefore, investigation of the expression and prognostic and immune relevance of NADPH oxidase would be significant to improve operability and prognosis.

NADPH oxidase (Nox) and dual oxidase (Duox) are evolutionarily conserved families with diverse seven members in human [16, 17] and are encoded by corresponding genes [18–20], namely *NOX1-5* and *DUOX1-2*. NADPH oxidases form a family of transmembrane proteins that generate reactive oxygen species (ROS) in the respiratory chain. Oxidation/reduction imbalance may contribute to the pathogenesis of a variety of human cancers [21]. The excessive production of ROS by NADPH oxidase may trigger tumorigenesis or progression induced by oxidative stress [22, 23]. Till date, specific isoforms have been identified in a variety of human malignancies, including colon [24, 25], gastric [26] and prostate [21] cancers. While specific roles of NADPH oxidases are yet to be determined in lung cancer, the current study aimed to investigate the expression, and prognostic and immune-related role, of this gene family in early and locally advanced stages of LUAD to provide clinical reference.

## Materials and methods

### Expression of NADPH oxidase in LUAD

Oncomine™ 4.5 Research Edition [27] (http://www.oncomine.org/), used for exploring NADPH oxidase expression, is a web-based database that contains data related to cancer microarray, for conducting genome-wide analyses of major cancer types and normal tissues, and for comparing transcriptome expression from the data analyzed; it currently contains 715 data sets (86,733 samples). In this study, we used the database to determine the mRNA expression of NADPH oxidase in LUAD and compared the mRNA levels between LUAD and normal controls; *P* = 0.05, fold change value > 1.5, top 10% genes were the threshold. Data entries from March 2021 to August 2021 were considered, and graphs were drawn with GraphPad Prism 7 software (GraphPad Software, Inc.).

### NADPH oxidase survival analysis in LUAD

Kaplan-Meier plotter [Lung Cancer] [28, 29] was used to assess the prognostic relevance of NADPH oxidase specifically expressed in LUAD. In our study, Affymetrix Identity (Jetset best Probe [30], as shown in Table S1) identified the available genes, and used median gene expression values to divide patient samples into high and low expression group; 95% confidence interval (CI) calculated log-rank P value and hazard ratio (HR), “Array quality control” and “Exclude biased array " were selected to obtain numerical results through univariate Cox regression analysis. Finally, the Kaplan-Meier survival curve (OS, Overall survival) was generated according to these parameters.

### Immunological correlation of NADPH oxidase

The immune correlation of NADPH oxidase was completed through the Tumor Immune Estimation Resource [31] (TIMER 2.0, timer.comp-genomics.org/), a web server for comprehensive analysis of tumor infiltrating immune cells, including The Cancer Genome Atlas (TCGA) of cancer genome maps from 32 cancer types, a total of 10,897 samples, and six subsets [32] of TIIC, including B cells, CD4 + T cells, CD8 + T cells, macrophages, neutrophils and dendritic cells. We investigated the significant NADPH oxidase mRNA expression data and tumor infiltration of six immune cell types in lung adenocarcinoma (LUAD).

### Patients and tissue samples

For this study, tissue microarray (TMA) chips for stage I-III LUAD were purchased, which included 15 paired tumor and adjacent samples (cDNA-HLugA030PG01; Shanghai OUTDO BIOTECH Co., Ltd.). The study was approved by the Ethics Committee of the First Hospital of Jiaxing, and informed consent was obtained from all subjects.

### RT-qPCR of NOX2 and NOX4

RNA from tissue samples was extracted with SYBR kit, and reverse transcribed with cDNA reverse transcription kit, according to the manufacturer’s instructions. The cDNA obtained by reverse transcription was diluted to 20ng/µl, and 2 µl of it was added to a 96-well plate for qPCR, the total reaction volume being 20 µl. NOX2 and NOX4 expression levels were normalized to that of internal glyceraldehyde-3-phosphate dehydrogenase (GAPDH) and determined by the 2^−△△Ct^ method.

### Statistical analysis

Relationship between their expression were compared using the paired T test analysis in tumor and adjacent tissues. Data were analyzed using GraphPad Prism 7 (GraphPad Software, Inc., L a Jolla, C A). Associations across stages and expressions was compared using the independent sample T test, and analysis was performed using the SPSS software package (SPSS Inc, Chicago, IL, USA, version 26.0). All tests were two-sided and P values < 0.05 were considered statistically significant.

## Results and discussion

**Fig. 1:**
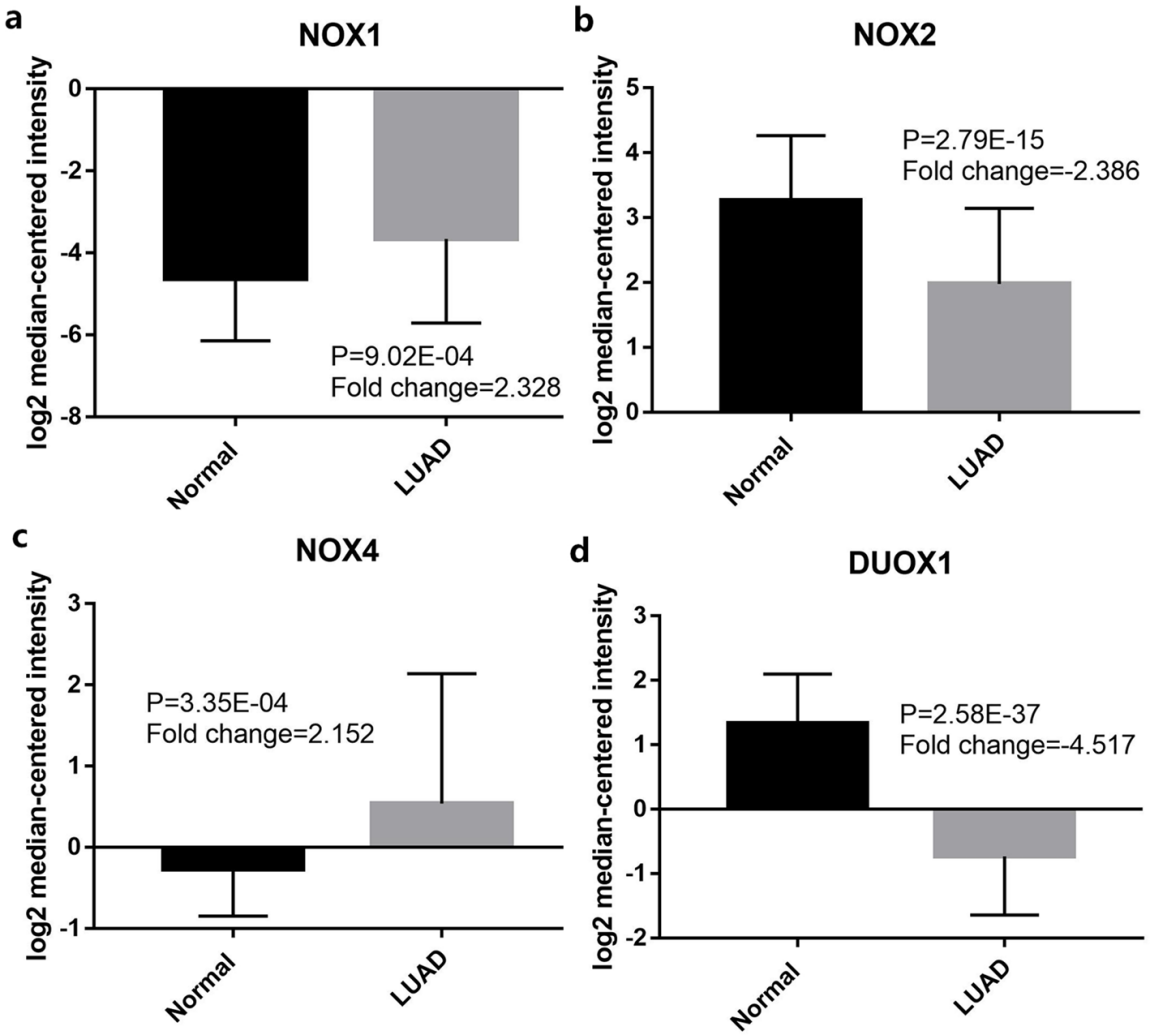
The analysis of NADPH oxidase in LUAD from Oncomine analysis. Column bar graph derived from gene expression data in normal (left plot) and cancer (right plot) tissue and plotted using Graphpad Prism 7 software. Y-axis represents Mean with Standard Deviation (M ± SD). **a** Comparison of NOX1 mRNA expression. **b** Comparison of NOX2 mRNA expression. **c** Comparison of NOX4 mRNA expression. **d** Comparison of DUOX1 mRNA expression. LUAD, Lung adenocarcinoma

Analysis of the Oncomine™ database revealed the expression of NADPH oxidase in patients with LUAD compared to that in normal. Column/bar graph in Fig. 1 represents the expression of each gene in tumors. The analysis demonstrated that, compared to normal, the expression levels of different NADPH oxidase mRNA in LUAD of different pathological types were significantly difference. Therefore, while NOX3, NOX5 and DUOX2 did not meet the conditions, NOX1 and NOX4 were over-expressed, and NOX2 and DUOX1 were under-expressed. Detailed databases [33–38] of NADPH oxidase expression based on gene rank are summarized in Table 1.

**Table 1:** The expression of NADPH oxidase in LUAD based on Gene Rank

	Fold Change	Dataset	Sample number		Total	*P*-value
			Normal	Cancer		
NOX1	2.328	Su Lung	30	27	57	9.02 × 10^− 14^
NOX2	−2.386	Selamat Lung	58	58	116	2.79 × 10^− 15^
NOX4	2.152	Garber Lung	39	5	44	3.35 × 10^− 4^
	1.502	Landi Lung	49	58	107	7.92 × 10^− 11^
	1.595	Su Lung	30	27	57	4.86 × 10^− 4^
	2.103	Hou Lung	65	45	110	3.70 × 10^− 7^
DUOX1	−4.517	Landi Lung	49	58	107	2.58 × 10^− 37^
	−6.348	Hou Lung	65	45	110	9.43 × 10^− 19^
	−5.812	Su Lung	30	27	57	1.82 × 10^− 11^
	−4.791	Okayama Lung	20	226	246	1.14 × 10^− 16^
	−4.210	Selamat Lung	58	58	116	1.20 × 10^− 26^

*LUAD* lung adenocarcinoma

**Fig. 2:**
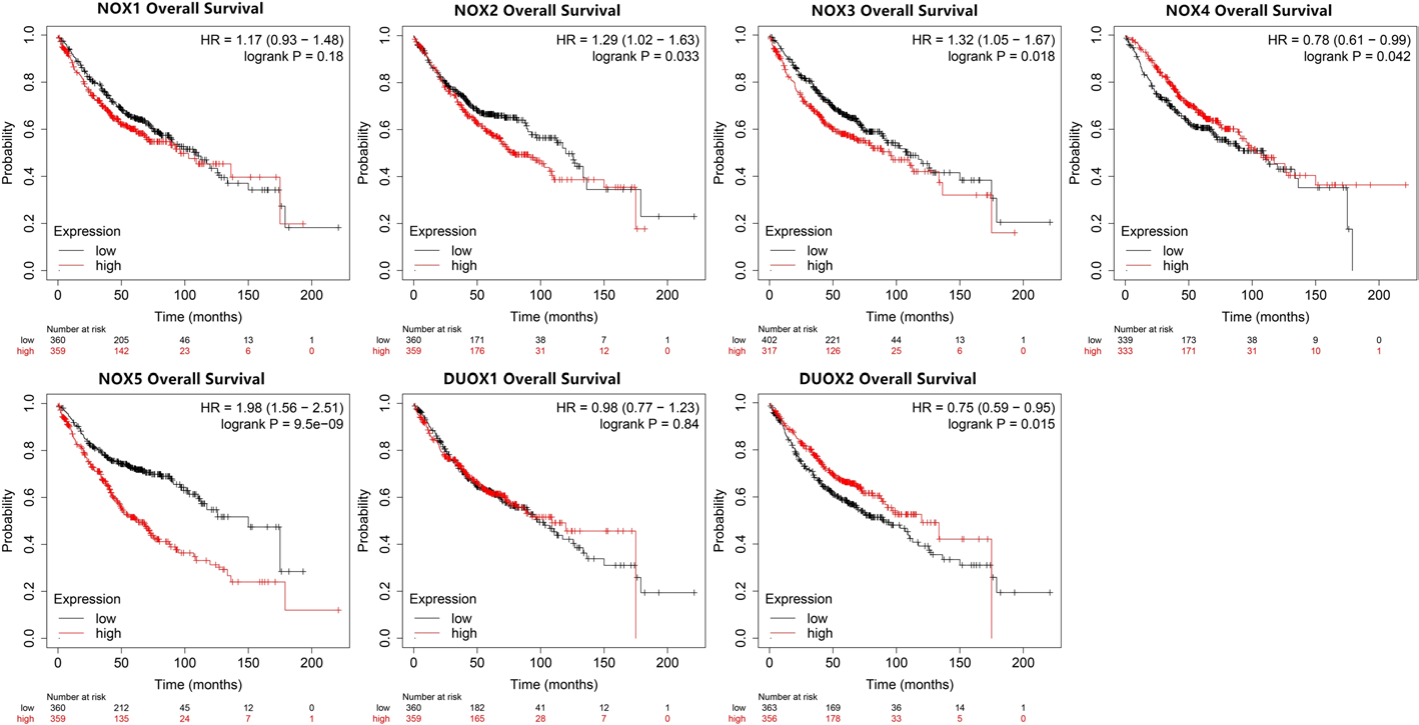
The prognostic value of NOX expression. Survival curves were plotted for LUAD patients(*n* = 672). Data was analyzed using Kaplan-Meier Plotter. Patients with expression above the median are indicated in red line, and patients with expressions below the median in black line. Abbreviation: HR, hazard ratio; CI, confidence interval

The prognostic value of NADPH oxidase mRNA expression was examined by Kaplan-Meier Plotter. (Kaplan Meier-plotter [Lung Cancer] 2015 version). The results in Fig. 2 show that NOX1 and DUOX1 mRNA expression did not correlate with high or low OS, with P values of 0.18 and 0.84, respectively; whereas overexpression of NOX2, NOX3 and NOX5 mRNA was associated with decreased OS; in contrast overexpression of NOX4 and DUOX2 mRNA predicted higher OS, with P values of 0.42 and 0.015, respectively (Table S2).

The associations between NADPH oxidase and clinicopathological characteristics of patients with LUAD, including stage, gender, smoking status, successful surgery and chemotherapy, were also examined. Grade and radiotherapy were not demonstrated to be associated with NADPH oxidase expression (data not shown). As indicated in Table S3, NOX2 and NOX5 were significantly associated with stage I while NOX5 was associated with stage II; no subtype was statistically significant in stage III. NOX2 and NOX5 were significantly associated with male patients, as shown in Table S4. Meanwhile, NOX2 and NOX5 were significantly associated with smoking patients (Table S5). NOX2 was significantly associated with only surgical margins negative, P value being 0.0054 (Table S6). Finally, NOX3 and DUOX2 were associated to prognosis upon chemotherapy (Table S7).

Using the TIMER database, we found the expression of NADPH oxidase mRNA in the LUAD immune microenvironment (B cells, CD8 + T cells, CD4 + T cells, macrophages, neutrophils and dendritic cells). Based on tumor purity, only NOX1, NOX2 and NOX4 expression was associated with infiltration level of immune cells (Figure S1). NOX2 expression was positively related to tumor purity and had positive correlation with infiltrating levels of all immune cells; NOX1 was positively related to infiltrating levels of B cells, CD4 + T cells, and dendritic cells while NOX4 was positively related to infiltrating levels of macrophages, neutrophils, and dendritic cells. Furthermore, we analyzed the survival cycle (tumor purity and stage), and found only NOX2 and NOX4 to be positively correlated with the prognosis of various immune cells in LUAD (P values were 0.043 and 0.017, respectively, *P* < 0.05), and others were not statistically significant, as shown in Figure S2.

**Fig. 3:**
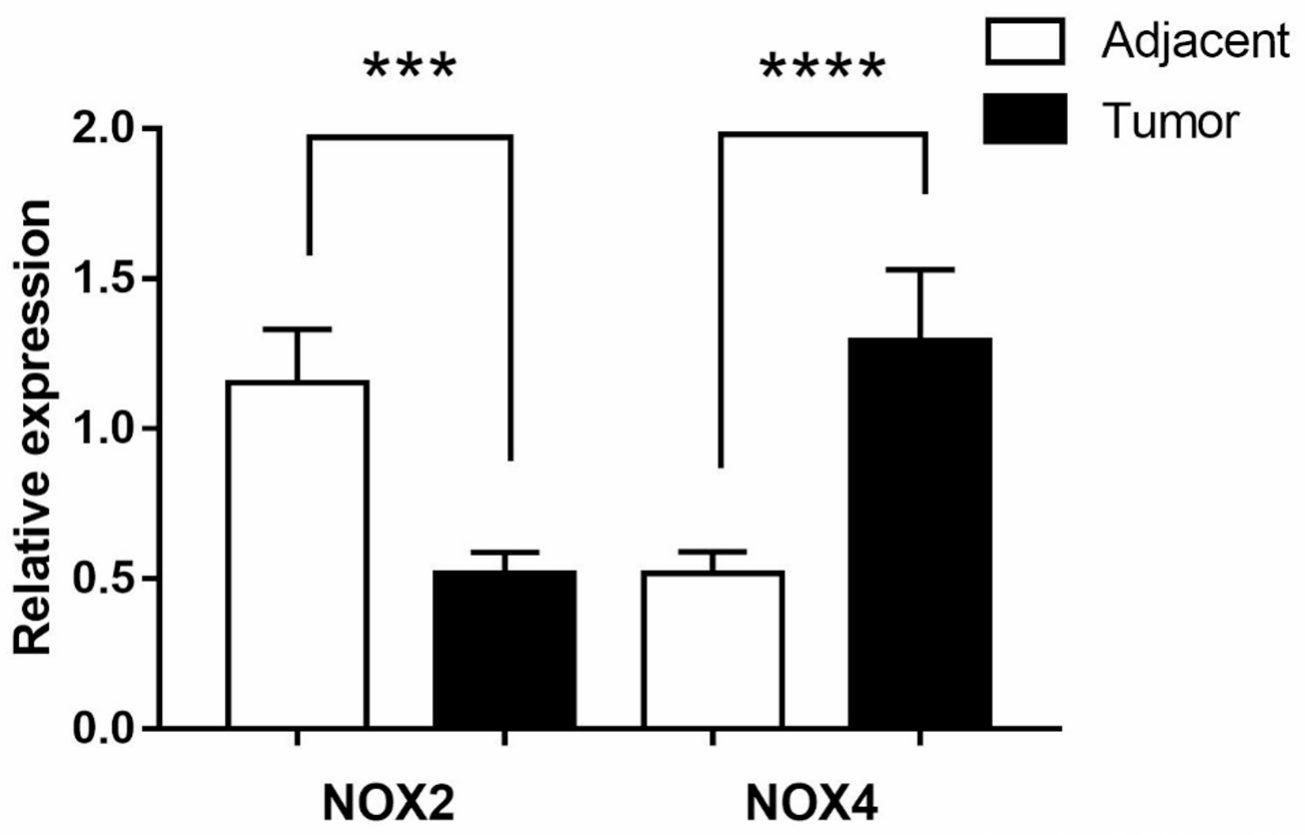
Relative mRNA expression of NOX2 and NOX4 by RT-qPCR; *** means Adjacent vs. Tumor in NOX2 statistical significance, *P* < 0.05; **** means Adjacent vs. Tumor in NOX4 statistical significance, *P* < 0.05. T-test was used to compare the two groups of paired samples

To examine NOX2 and NOX4 mRNA expression and the relationship between expression level and LUAD stage, tissue microarray assay (TMA) was performed. We explored the general clinical characteristics and original pathological reports of 15 pairs of patients with LUAD, as detailed in Table 2. Using RT-qPCR quantitative expression analysis, the expression of NOX2 mRNA was found to be lower than that of NOX4 in LUAD, as shown in Fig. 3. Paired T test was used to analyze and compare the differences in expression of NOX2 and NOX4 mRNA in tumor and adjacent tissues of LUAD; P values of 0.0033 and 0.0096, respectively, implied statistically significant differences. Furthermore, at different stages, NOX4 was found to be higher than in adjacent tissues in stage I LUAD(*P* = 0.045 < 0.05), and there was no significant difference between stage II and stage III or even between I + II and III; however, difference in NOX2 was not statistically significant. The relationships between the expression levels and stage are presented in Table 3.

**Table 2:** Clinical characteristics of 15 pairs of LUAD patients for RT-qPCR

Tissue types	Gander	Age(Y)	Size(cm)	Site	Tumor(T)	Lymph Node(*N*)	TNM (AJCC 7 edition*)
Tumor/Adjacent	Female	48	5*4*5	Right lower lobe	T2a	N0	IB
Tumor/Adjacent	Female	71	3*2.5*2	Right upper lobe	T1b	N2	IIIA
Tumor/Adjacent	Male	74	6*6*5	Right lower lobe	T2b	N0	IIA
Tumor/Adjacent	Male	63	3*3*2.8	Right upper lobe	T1b	N1	IIA
Tumor/Adjacent	Female	59	14*9*6	Left lower lobe	T3	N0	IIB
Tumor/Adjacent	Female	54	3*2*2	Left lower lobe	T1b	N1	IIA
Tumor/Adjacent	Male	63	5*4*2.5	Left upper lobe	T2a	N0	IB
Tumor/Adjacent	Male	64	5*3*3	Right lower lobe	T2a	N0	IB
Tumor/Adjacent	Female	68	5*4*4	Left upper lobe	T2a	N1	IIA
Tumor/Adjacent	Female	81	3*3*2	Right upper lobe	T1b	N0	IA
Tumor/Adjacent	Female	65	6*4*2.5	Right middle lobe	T2b	N1	IIB
Tumor/Adjacent	Female	50	3*2.5*2	Right lower lobe	T1b	N2	IIIA
Tumor/Adjacent	Male	60	4*3*3	Left upper lobe	T2a	N1	IIA
Tumor/Adjacent	Male	54	4*3.5*3	Right lower lobe	T2a	N2	IIIA
Tumor/Adjacent	Male	77	6*5*5	Right lower lobe	T2b	N0	IIA

*LUAD* lung adenocarcinoma, *RT-qPCR* Real Time Quantitative Polymerase Chain Reaction; *Ref: AJCC Cancer Stating Manual 7th Edition, American Joint Committee on Cancer, ISBN 978-0-387–88440-0. Springer, New York, Dordrecht, Heidelberg, London

**Table 3:** Relationship between the expression of NOX2 and NOX4 in different LUAD stage

Stage		NOX2		NOX4		^a^T value	^a^*P* value	^b^T value	^b^*P* value
		Adjacent	Tumor	Adjacent	Tumor				
IA + IB		21.74 ± 0.19	23.17 ± 0.16	26.79 ± 0.19	25.73 ± 0.17	−1.437	0.201	2.533	0.045
IIA + IIB		22.24 ± 0.20	22.76 ± 0.14	26.26 ± 0.19	25.08 ± 0.21	−0.747	0.467	1.674	0.116
IIIA		21.37 ± 0.11	22.71 ± 0.14	26.72 ± 0.10	24.63 ± 0.14	−1.063	0.348	1.866	0.135
I + II vs. III	^c^T value		0.201		0.814				
	^c^P value		0.844		0.430				

*LUAD* lung adenocarcinoma, *SD* standard deviation, *T value* Independent sample T test between two groups, represents the difference is statistically significant, *P* < 0.0

^a^means Adjacent vs. Tumor in NOX2; ^b^ means Adjacent vs. Tumor in NOX4; ^c^ means I + II vs. III in *NOX2* or *NOX4* Tumor

NADPH oxidases generate a defined burst of reactive oxygen species (ROS) as a controlled response to particular stimuli, and hence, should be considered as significant contributors in redox signaling. NOXs may participate in the multi-step development of human cancers via the generation of ROS [39]. Since some anticancer therapeutics use selective oxidative stress as a pro-apoptotic approach, NADPH oxidases can have an important effect on the incidence and treatment of cancer. Till date, NOX isoforms have been identified in a variety of human malignancies, including colon [24, 25], gastric [26] and prostate [21] cancers, and have been studied in considerable detail in lung cancer.

NOX1 was initially designated NOH-1 (NADPH oxidase homolog-1) [40] and participates in the NOX1/Ras/ERK-MAPK pathway mediating the transcription of vascular endothelial growth factor (VEGF), a key mediator of tumor angiogenesis [41]. Disrupting ROS homeostasis or inhibiting NOX1 reduces tumor growth and angiogenesis in vivo [42]. In our study, NOX1 was similarly overexpressed in LUAD. NOX3 and NOX1 are the most closely related isoforms among phagocyte NADPH oxidases, sharing 60% sequence homology with NOX2 [43]. NOX3 exhibits low abundance in lung tissue, and its function remains unclear [44]. This study reveals that NOX3 overexpression correlates with poor prognosis in lung adenocarcinoma. Some studies have suggested that NOX5/ROS axis-mediated tumor/stroma crosstalk, involving prostate cancer cells [45] and colon cancer cells [46] provides applicable evidence for tumor treatment with chemotherapy. But there is no evidence for this in lung cancer, especially lung adenocarcinoma. Our study revealed that high levels of NOX5 mRNA in early-stage LUAD (Stages I and II) predicted shorter overall survival, particularly in male smokers. DUOX1 and DUOX2 were initially discovered in human thyroid tissue and are closely associated with thyroid cancer [47]. Current understanding of the regulatory mechanisms governing DUOX genes remains incomplete. Existing studies indicate that these two proteins play significant roles in lung cancer by downregulating promoter methylation [48]. The absence of DUOX1 promotes overall reduction in EGFR cysteine, thereby participating in carcinogenesis through EGFR gene regulation [49]. We observed that DUOX1 exhibits low expression in LUAD, while DUOX2 mRNA shows high expression characteristics; however, we are unable to provide further evidence to support this finding.

NOX2 and NOX4 are key members of the NADPH oxidase family. NOX2 synthesizes superoxide anion and NOX4 hydrogen peroxide, located in cell membrane and cytoplasm respectively [50]. A previous study had showed NOX2 inhibition to promote NK cell-mediated tumor cell clearance, thus supporting the promotion of metastasis by NOX2 via immunosuppression [51].

NOX4 is a distinct member of the NADPH oxidase family that constitutively generates hydrogen peroxide (H_2_O_2_) rather than superoxide, and exhibits remarkable context-dependent functions across different tissues and disease states [52, 53]. NOX4 is a unique member of the NADPH oxidase family, characterized by its dual function of continuously generating hydrogen peroxide (H_2_O_2_) rather than superoxide. This dual role is increasingly recognized as a hallmark of redox-sensitive signaling, exhibiting significant context-dependent functionality across different tissues and disease states [54]. In LUAD, the hypoxic microenvironment may activate a HIF-1α-NOX4 positive feedback loop, wherein H_2_O_2_ generated by NOX4 promotes hypoxic adaptation by stabilizing HIF-1α. Second, the high prevalence of EGFR mutations in lung adenocarcinoma may render cells susceptible to NOX4 signaling regulation; NOX4 inactivates protein tyrosine phosphatases (PTPs) via reactive oxygen species (ROS), thereby sustaining EGFR phosphorylation and amplifying downstream oncogenic pathways [55, 56]. Future studies should utilize LUAD models with defined EGFR mutation status under controlled oxygen concentration conditions. Based on these observations, we hypothesized that both NOX2 and NOX4 paly active roles in LUAD. The results of our study indicated that under-expression of NOX2 mRNA and over-expression of NOX4 mRNA in tumor tissues compared to that in adjacent tissues. Due to the small sample size and other restrictions, our study found only NOX4 to be highly expressed and show poor prognosis in stage I LUAD.

Regarding immune responses, this study found that NOX2 and NOX4 expression positively correlated with tumor purity and with the infiltration levels of multiple immune cell types. Extensive research [57] indicates that NOX2 and NOX4, as oxidases maintaining self-tolerance, can serve as targetable immune checkpoints in lung cancer, particularly since NOX4 is closely associated with poor prognosis. Liu et al. knocked down NOX4 in lung adenocarcinoma cell lines (e.g., A549, H1975) using shRNA or CRISPR technology to evaluate its effects on cell proliferation, migration, and PD-L1 expression. Recent studies also provide theoretical support for combining NOX4 inhibition with immunotherapy [56, 58, 59]: In NSCLC, elevated NOX4 levels promote tumorigenesis and confer EGFR-TKI resistance by enhancing the IL-8/PD-L1 signaling pathway; GKT137831 synergistically suppressed tumor growth and angiogenesis in NSCLC models when combined with the anti-PD-L1 antibody durvalumab or the anti-angiogenic agent bevacizumab; NOX4-derived reactive oxygen species (ROS) activate the NF-κB signaling pathway, thereby upregulating PD-L1 and suppressing T cell function. In a follow-up study, we aim to explore the targeted inhibitor of NADPH oxidase and identify a marker of immunotherapy therefrom.

## Conclusion

In summary, the Oncomine™ database was used in this study to examine gene expression in LUAD, and the prognostic values of genes were analyzed by Kaplan-Meier analysis. The results indicated over-expressed NOX4 to be associated with lower prognosis; NOX2 was found to be under-expressed in LUAD. Both NOX2 and NOX4 expression was positively related to tumor purity and had positive correlation with infiltrating levels of immune cells in TIMER. The data reflected the potential association of NADPH oxidase in early and locally advanced lung adenocarcinoma. Our data suggested that the combination of NOX4 suppression, NOX2 depression, and immunotherapy would improve clinical outcome in these tumors. However, further analysis of its post-transcriptional regulation and immune regulation in the microenvironment would be required in future, so that it can be the focus of tumor immunotherapy. The limitations of this study have prompted us to further explore the physiological and biochemical mechanisms of advanced lung adenocarcinoma, especially in tumor metastasis, gene targeted therapy, immunotherapy, and other physiological and biochemical mechanisms.

## Supplementary Information

Supplementary Material 1.

## Data Availability

The datasets analyzed in the present study are available in the Oncomine TM database (https://www.oncomine.org/resource) and the Kaplan Meier-plotter [Lung Cancer] (http://kmplot.com/analysis/index.php? p=service&cancer=lung) and the TMIER(http://timer.comp-genomics.org/).
