# Supplementary material for: The prognostic and immunological role of NADPH oxidase in early and locally advanced lung adenocarcinoma
Source: Discov Oncol. 2026 Jun 10;17:1186. doi: 10.1007/s12672-026-05404-3 (PMC13478044; doi:10.1007/s12672-026-05404-3)
Supplement: Supplementary file 1 [file 12672_2026_5404_MOESM1_ESM.docx]

**Supplementary Tables**

Table S1. Affymetrix ID of *NOXs* genes in the Kaplan-Meier plotter.

| NOXs | Affymetrix ID (Jetset best probe set) |
| --- | --- |
| NOX1 | 207217_s_at |
| NOX2 | 203923_s_at |
| NOX3 | 221089_at |
| NOX4 | 236843_at |
| NOX5 | 220641_at |
| DUOX1 | 219597_s_at |
| DUOX2 | 219727_at |

NOX, NADPH (Nicotinamide Adenine Dinucleotide Phosphate) oxidase

Table S2. Prognosis of NADPH oxidase in LUAD patients.

|  | Case-low | Case-high | HR(95%CI) | P value |
| --- | --- | --- | --- | --- |
| NOX1 | 360 | 359 | 1.17(0.93-1.48) | 0.18 |
| NOX2 | 360 | 359 | 1.29(1.02-1.63) | 0.033 |
| NOX3 | 402 | 317 | 1.32(1.05-1.67) | 0.018 |
| NOX4 | 339 | 333 | 0.78(0.61-0.99) | 0.042 |
| NOX5 | 360 | 359 | 1.98(1.56-2.51) | 9.5E-09 |
| DUOX1 | 360 | 359 | 0.98(0.77-1.23) | 0.84 |
| DUOX2 | 363 | 356 | 0.75(0.59-0.95) | 0.015 |

Abbreviation: LUAD, lung adenocarcinoma; HR: hazard ratio; CI: confidence interval; Cases-low/high: patient number of low/high expression of the corresponding gene.

Table S3. Correlation of NADPH oxidase with Stage of LUAD patients.

| Gene | Stage | Case-low | Case-high | HR(95%CI) | logrank P |
| --- | --- | --- | --- | --- | --- |
| NOX1 | I | 186 | 184 | 0.95(0.65-1.40) | 0.80 |
|  | II | 69 | 67 | 1.55(0.96-2.52) | 0.072 |
|  | III | 12 | 12 | 0.76(0.26-2.22) | 0.62 |
| NOX2 | I | 185 | 185 | 2.00(1.33-3.00) | 6.2E-04 |
|  | II | 68 | 68 | 0.92(0.57-1.50) | 0.75 |
|  | III | 12 | 12 | 1.65(0.60-4.59) | 0.33 |
| NOX3 | I | 233 | 137 | 0.90(0.60-1.36) | 0.63 |
|  | II | 83 | 53 | 1.41(0.87-2.30) | 0.16 |
|  | III | 12 | 12 | 0.88(0.33-2.40) | 0.81 |
| NOX4 | I | 174 | 172 | 0.69(0.46-1.03) | 0.067 |
|  | II | 61 | 57 | 0.66(0.39-1.11) | 0.12 |
|  | III | 11 | 10 | 0.99(0.30-3.27) | 0.99 |
| NOX5 | I | 185 | 185 | 2.72(1.80-4.12) | 8.0E-07 |
|  | II | 68 | 68 | 1.85(1.13-3.03) | 0.013 |
|  | III | 13 | 11 | 1.37(0.48-3.93) | 0.55 |
| DUOX1 | I | 185 | 185 | 0.84(0.57-1.24) | 0.38 |
|  | II | 68 | 68 | 1.54(0.95-2.50) | 0.079 |
|  | III | 12 | 12 | 1.18(0.42-3.29) | 0.76 |
| DUOX2 | I | 185 | 185 | 0.74(0.50-1.09) | 0.12 |
|  | II | 69 | 67 | 1.10(0.68-1.77) | 0.71 |
|  | III | 12 | 12 | 0.41(0.15-1.14) | 0.079 |

Abbreviation: LUAD, lung adenocarcinoma; HR: hazard ratio; CI: confidence interval; Cases-low/high: patient number of low/high expression of the corresponding gene.

Table S4. Correlation of NADPH oxidase with gender in LUAD patients.

| Gene | Gender | Case-low | Case-high | HR(95%CI) | logrank P |
| --- | --- | --- | --- | --- | --- |
| NOX1 | Female | 159 | 158 | 1.35(0.92-1.96) | 0.12 |
|  | Male | 172 | 172 | 0.77(0.55-1.06) | 0.11 |
| NOX2 | Female | 158 | 159 | 1.26(0.86-1.84) | 0.24 |
|  | Male | 172 | 172 | 1.50(1.08-2.09) | 0.015 |
| NOX3 | Female | 183 | 134 | 1.45(0.99-2.11) | 0.054 |
|  | Male | 218 | 162 | 0.97(0.69-1.37) | 0.86 |
| NOX4 | Female | 146 | 140 | 0.70(0.47-1.05) | 0.086 |
|  | Male | 167 | 161 | 0.82(0.59-1.15) | 0.25 |
| NOX5 | Female | 158 | 159 | 1.91(1.29-2.82) | 9.5E-04 |
|  | Male | 173 | 171 | 1.48(1.07-2.06) | 0.017 |
| DUOX1 | Female | 159 | 158 | 1.41(0.96-2.06) | 0.078 |
|  | Male | 174 | 170 | 0.74(0.53-1.03) | 0.078 |
| DUOX2 | Female | 159 | 158 | 0.82(0.56-1.20) | 0.30 |
|  | Male | 177 | 167 | 0.67(0.48-0.93) | 0.017 |

Abbreviation: LUAD, lung adenocarcinoma; HR: hazard ratio; CI: confidence interval; Cases-low/high: patient number of low/high expression of the corresponding gene.

Table S5. Correlation of NADPH oxidase with smoking status of LUAD patients.

| Gene | Smoking status | Case-low | Case-high | HR(95%CI) | logrank P |
| --- | --- | --- | --- | --- | --- |
| NOX1 | Smoked | 127 | 119 | 1.16(0.73-1.85) | 0.54 |
|  | Never smoked | 103 | 102 | 2.86(1.56-5.24) | 3.8E-04 |
| NOX2 | Smoked | 123 | 123 | 1.79(1.10-2.90) | 0.017 |
|  | Never smoked | 72 | 71 | 2.35(0.97-5.67) | 0.051 |
| NOX3 | Smoked | 145 | 101 | 0.98(0.61-1.57) | 0.92 |
|  | Never smoked | 100 | 43 | 0.88(0.36-2.13) | 0.78 |
| NOX4 | Smoked | 118 | 113 | 0.75(0.46-1.22) | 0.25 |
|  | Never smoked | 71 | 69 | 1.91(0.81-4.50) | 0.13 |
| NOX5 | Smoked | 123 | 123 | 2.05(1.26-3.32) | 0.003 |
|  | Never smoked | 76 | 67 | 1.45(0.65-3.24) | 0.36 |
| DUOX1 | Smoked | 124 | 122 | 0.84(0.53-1.34) | 0.47 |
|  | Never smoked | 72 | 71 | 0.79(0.36-1.78) | 0.57 |
| DUOX2 | Smoked | 123 | 123 | 0.69(0.43-1.11) | 0.12 |
|  | Never smoked | 73 | 70 | 0.90(0.41-2.02) | 0.81 |

Abbreviation: LUAD, lung adenocarcinoma; HR: hazard ratio; CI: confidence interval; Cases-low/high: patient number of low/high expression of the corresponding gene.

Table S6. Correlation of NADPH oxidase with only surgical margins negative in LUAD patients.

| Gene | Case-low | Case-high | HR(95%CI) | logrank P |
| --- | --- | --- | --- | --- |
| NOX1 | 103 | 101 | 1.2 (0.59-2.46) | 0.62 |
| NOX2 | 102 | 102 | 2.99(1.33-6.72) | 0.0054 |
| NOX3 | 115 | 89 | 1.24(0.61-2.54) | 0.55 |
| NOX4 | 102 | 102 | 1.18(0.57-2.41) | 0.66 |
| NOX5 | 107 | 97 | 1.52(0.74-3.13) | 0.26 |
| DUOX1 | 102 | 102 | 0.55(0.26-1.15) | 0.11 |
| DUOX2 | 102 | 102 | 0.56(0.27-1.17) | 0.12 |

Abbreviation: LUAD, lung adenocarcinoma; HR: hazard ratio; CI: confidence interval; Cases-low/high: patient number of low/high expression of the corresponding gene.

Table S7. Correlation of NADPH oxidase with Chemotherapy of LUAD patients.

| Gene | Chemotherapy | Case-low | Case-high | HR(95%CI) | logrank P |
| --- | --- | --- | --- | --- | --- |
| NOX1 | No | 10 | 11 | 0.59 (0.15-2.32) | 0.44 |
|  | Yes | 18 | 18 | 2.46(0.65-9.32) | 0.17 |
| NOX2 | No | 10 | 11 | 1.42(0.37-5.41) | 0.60 |
|  | Yes | 18 | 18 | 1.78(0.50-6.36) | 0.37 |
| NOX3 | No | 10 | 11 | 0.72(0.18-2.93) | 0.65 |
|  | Yes | 19 | 17 | 0.27(0.07-1.02) | 0.043 |
| NOX4 | No | 6 | 5 | 0.71(0.10-5.30) | 0.74 |
|  | Yes | 10 | 9 | 4.65 (0.48-45.39) | 0.15 |
| NOX5 | No | 10 | 11 | 4.92(0.83-29.38) | 0.063 |
|  | Yes | 18 | 18 | 2.16(0.63-7.45) | 0.21 |
| DUOX1 | No | 10 | 11 | 0.88(0.24-3.31) | 0.86 |
|  | Yes | 18 | 18 | 2.01(0.58-6.99) | 0.26 |
| DUOX2 | No | 10 | 11 | 0.69(0.17-2.78) | 0.60 |
|  | Yes | 18 | 18 | 0.13(0.02-0.70) | 0.0073 |

Abbreviation: LUAD, lung adenocarcinoma; HR: hazard ratio; CI: confidence interval; Cases-low/high: patient number of low/high expression of the corresponding gene.

**Supplementary Figures**

Figure S1 Correlation between NOX and immune cells in LUAD.

Figure S2 Correlation between NOX and immune cells based on tumor purity and stage in LUAD.
